# Supplementary material for: Pooling quantitative MRI data: A multi-protocol study of healthy subcortical aging
Source: Imaging Neurosci (Camb). 2026 Jul 31;4:IMAG.a.1315. doi: 10.1162/IMAG.a.1315 (PMC13439991; doi:10.1162/IMAG.a.1315)
Supplement: Supplementary file 1 [file IMAG.a.1315_supp.pdf]

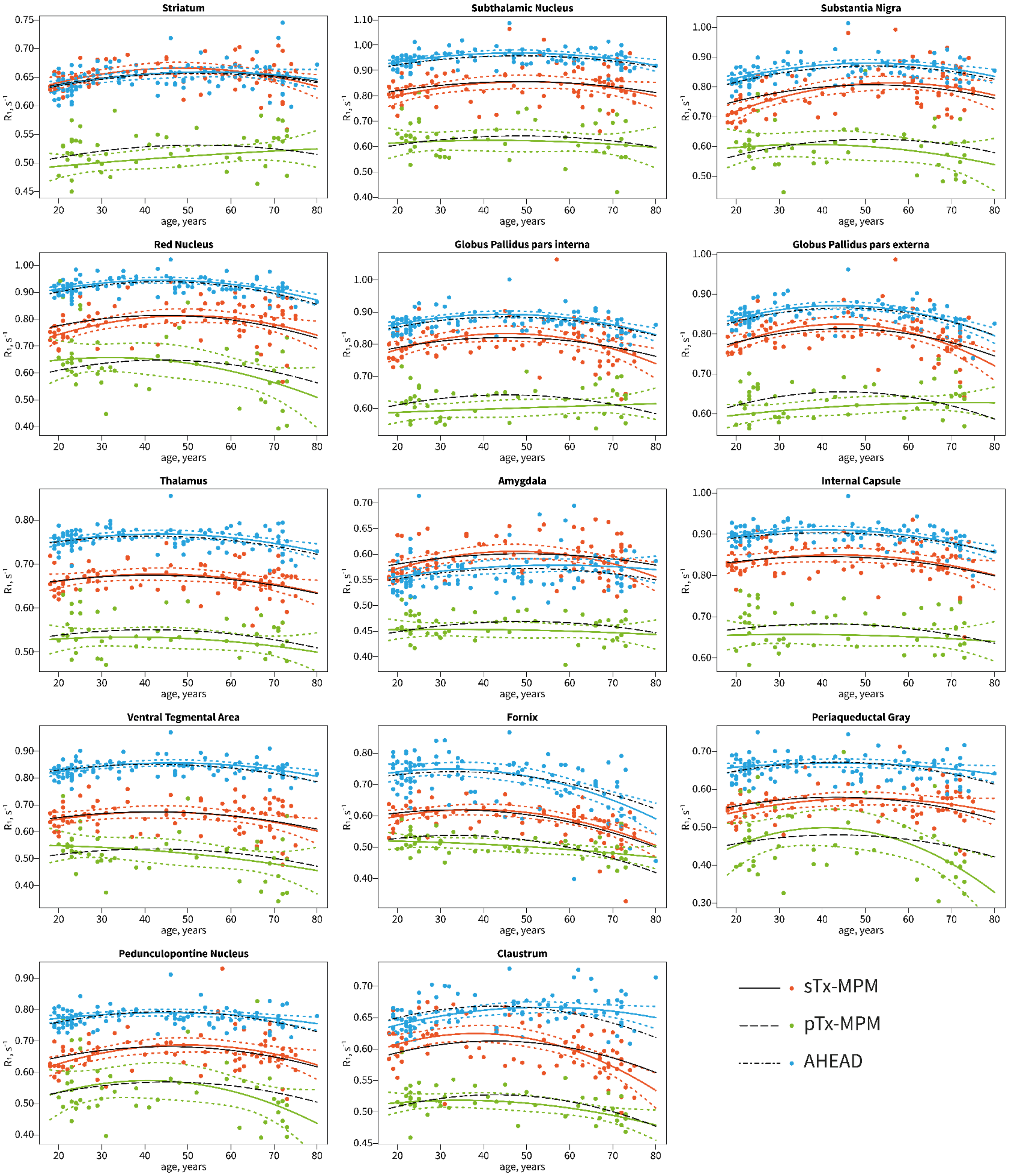

Figure S1 Age dependence of median R1 values measured in the 14 subcortical ROI measured in three datasets (dots of different colors). Each graph shows age dependence models that were fit for each of the protocol types presented as lines.

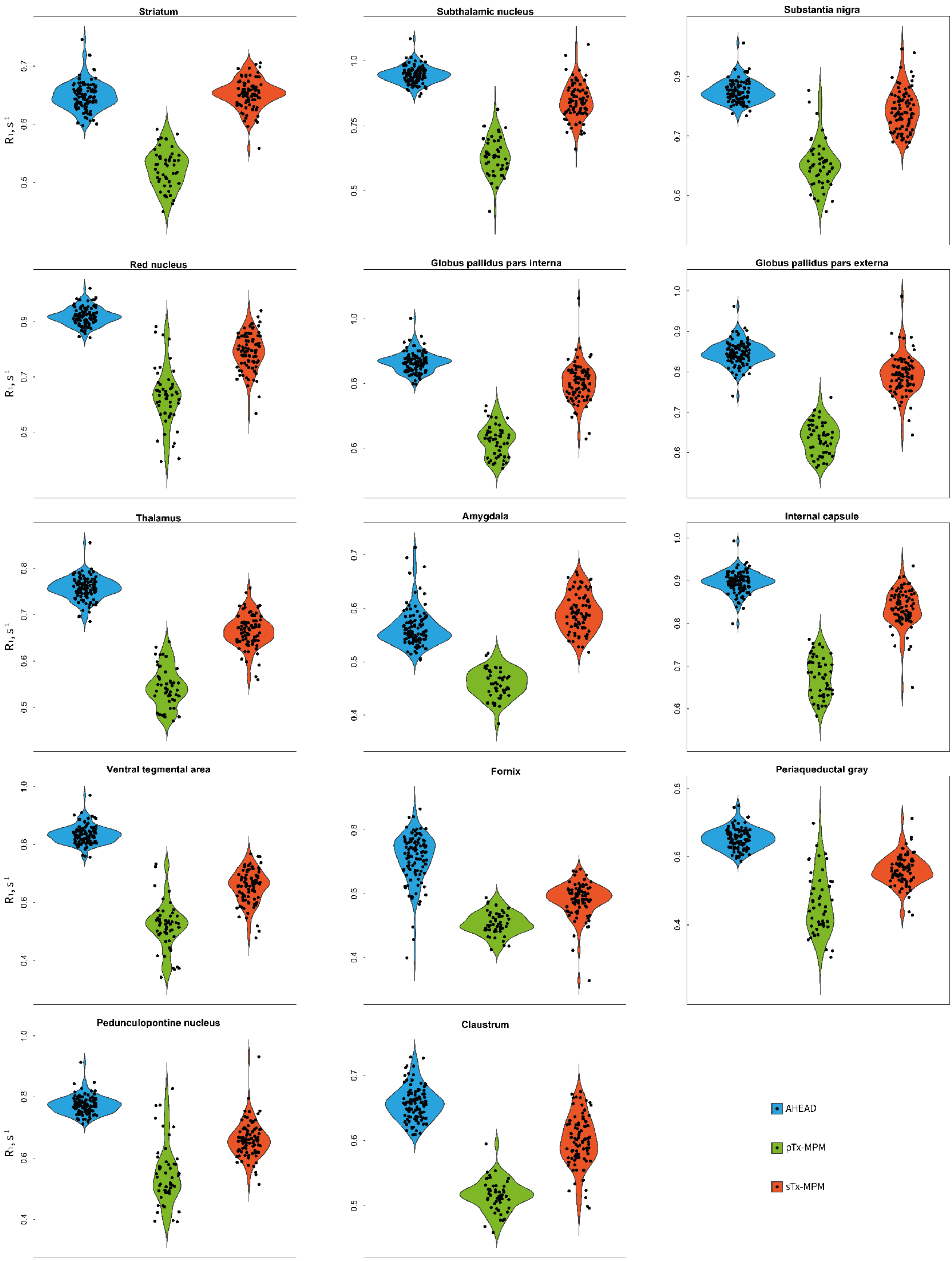

Figure S2 Protocol-stratified median R1 values measured in the 14 subcortical ROI. Graphs show data age-adjusted to the pooled dataset median age (45 years).

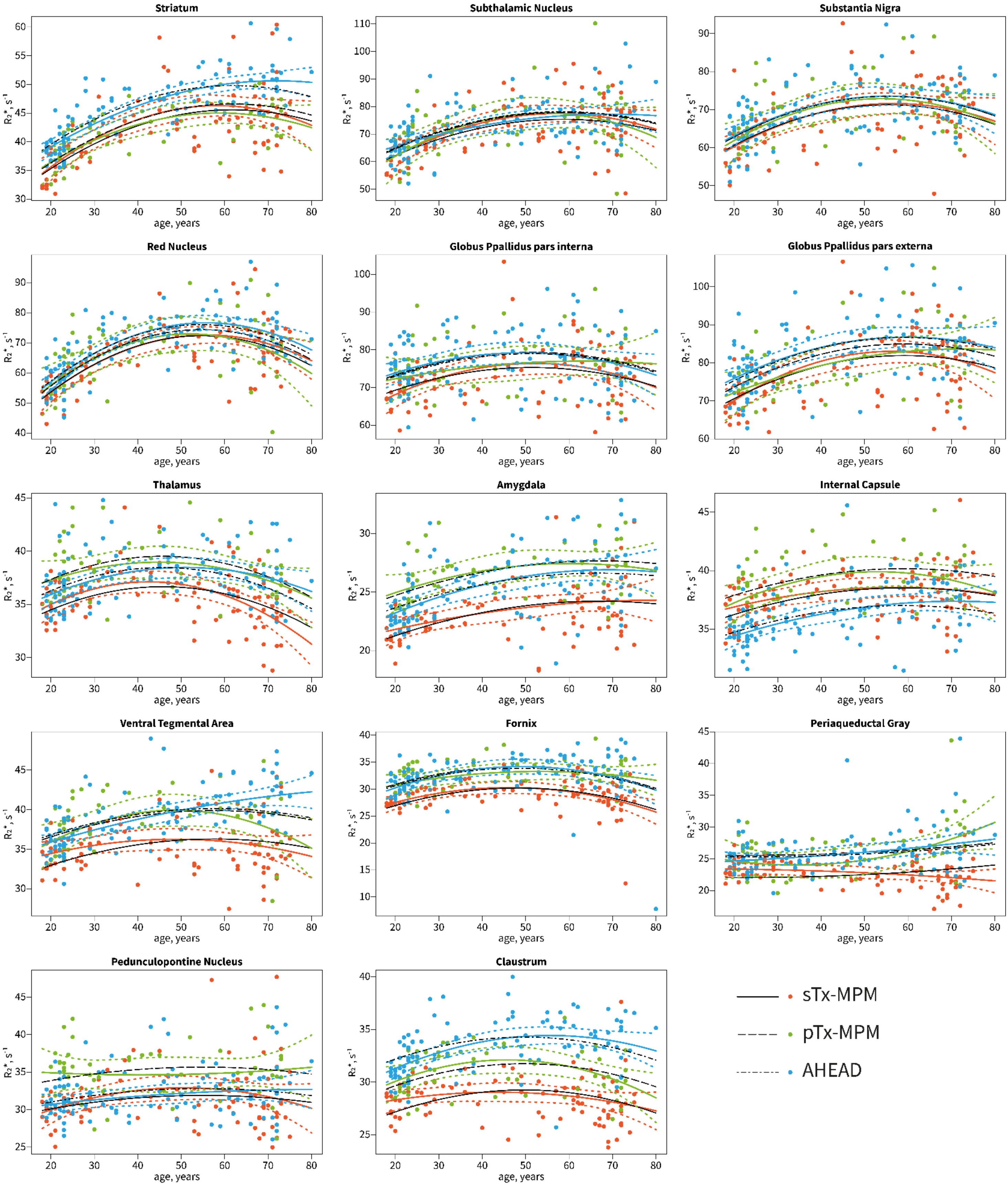

Figure S3 Age dependence of median R2 values measured in the 14 subcortical ROI (dots of different colors). Each graph shows age dependence models that were fit for each of the protocol types presented as lines.*

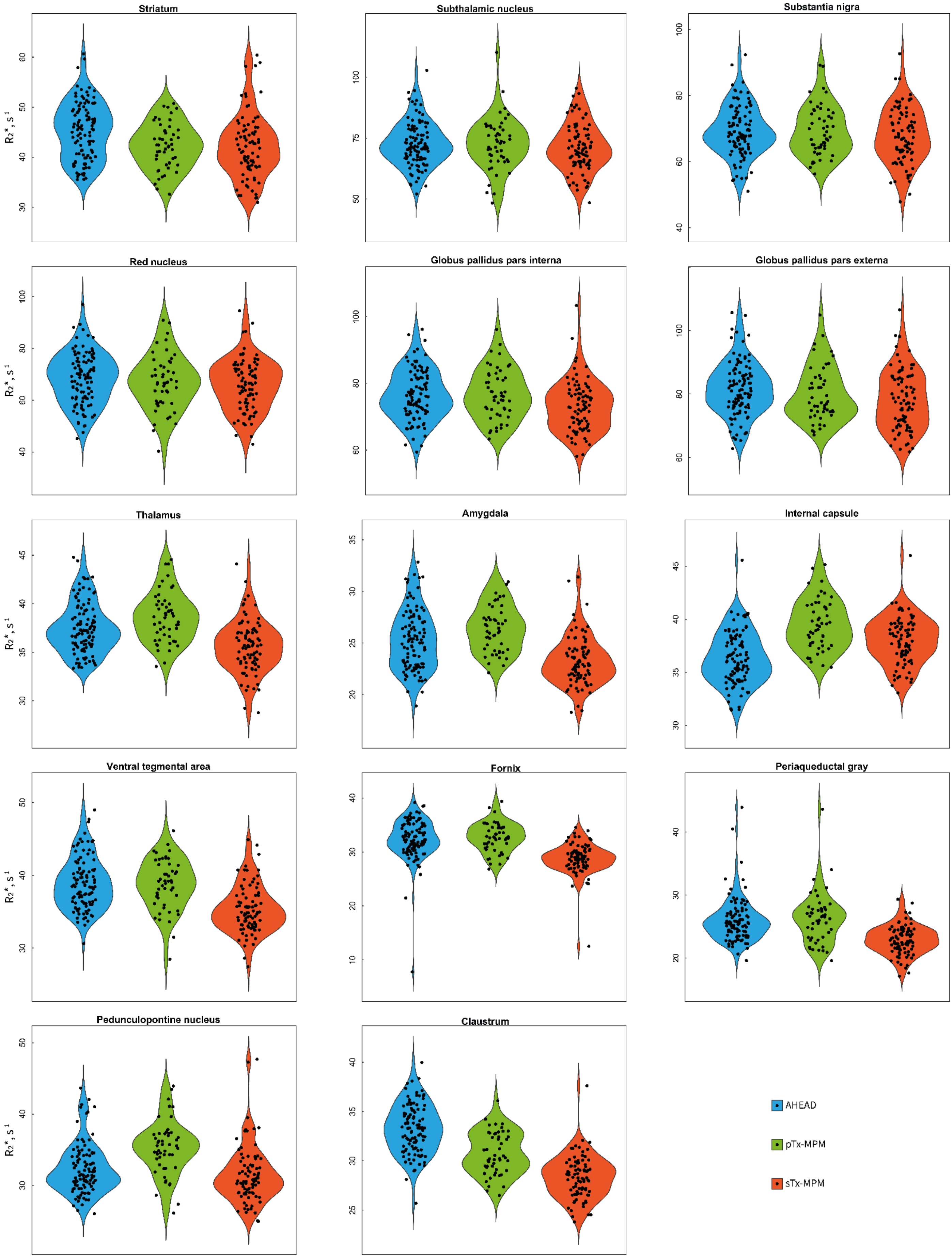

Figure S4 Protocol-stratified median R2 values measured in the 14 subcortical ROI. Graphs show data age-adjusted to the pooled dataset median age (45 years).*

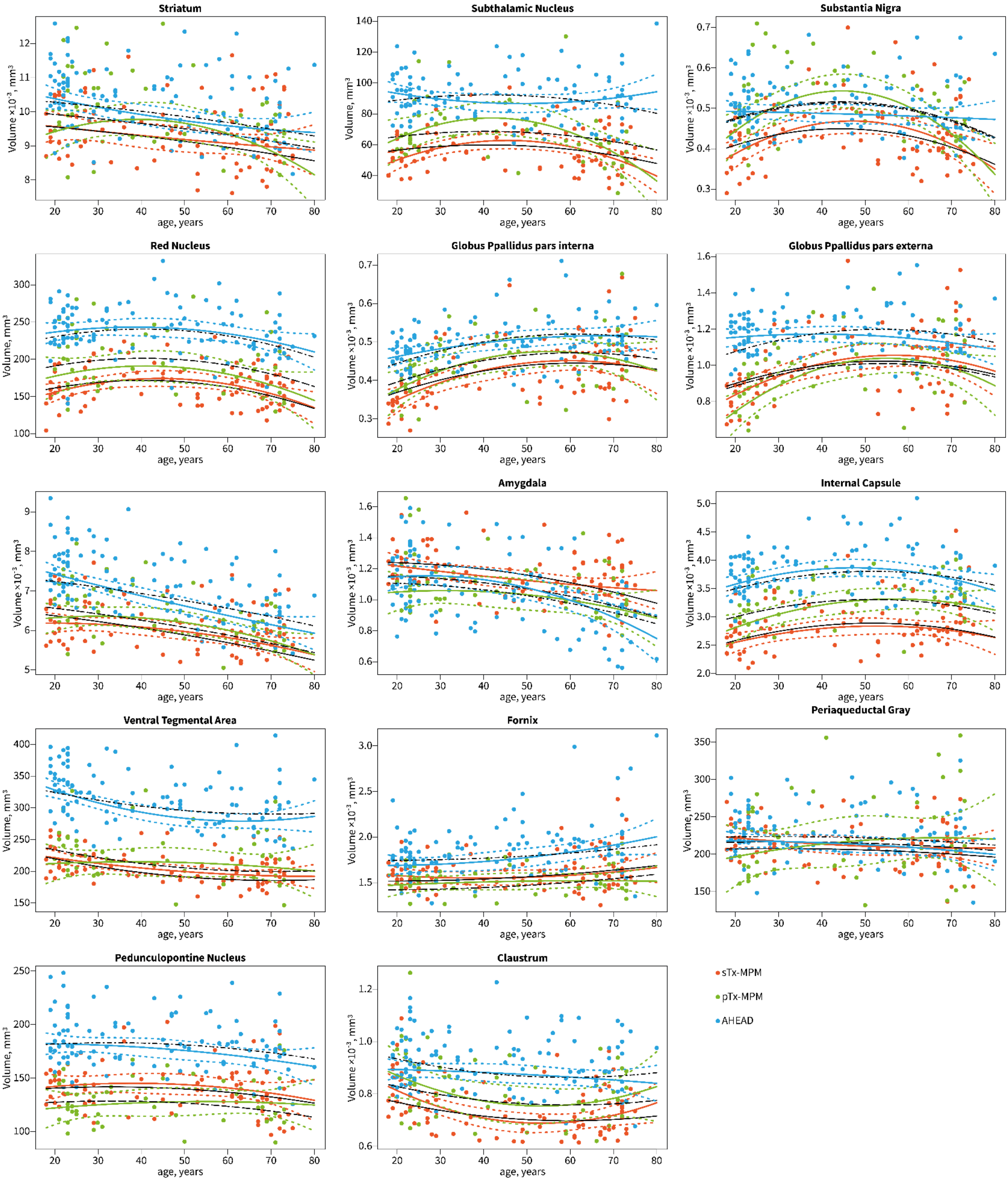

Figure S5 Age dependent volume measurements of the 14 subcortical ROI. Each graph shows age dependence models that were fit for each of the protocol types presented as lines.

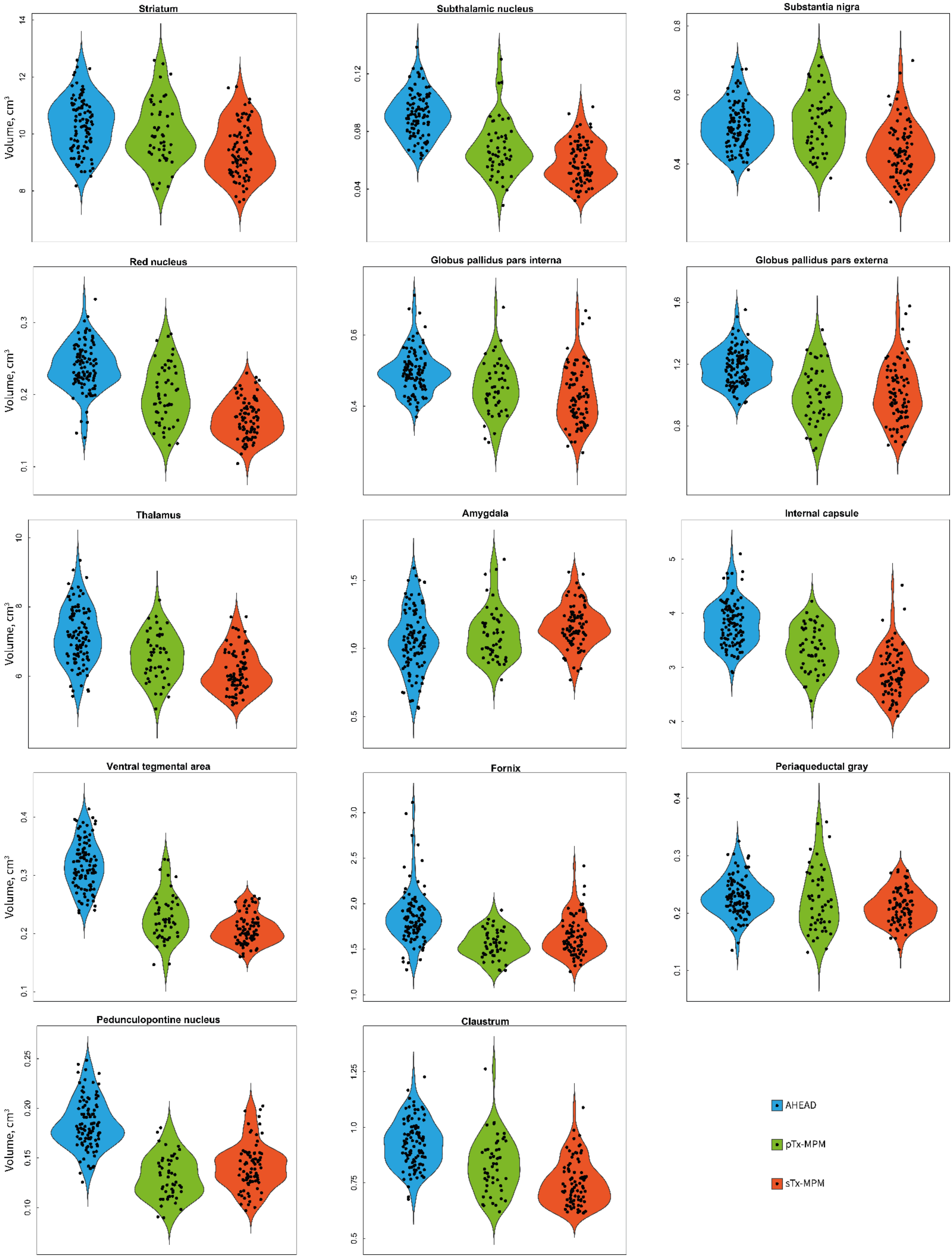

Figure S6 Protocol-stratified volume measurements of the 14 subcortical ROI. Graphs show data age-adjusted to the pooled dataset median age (45 years).

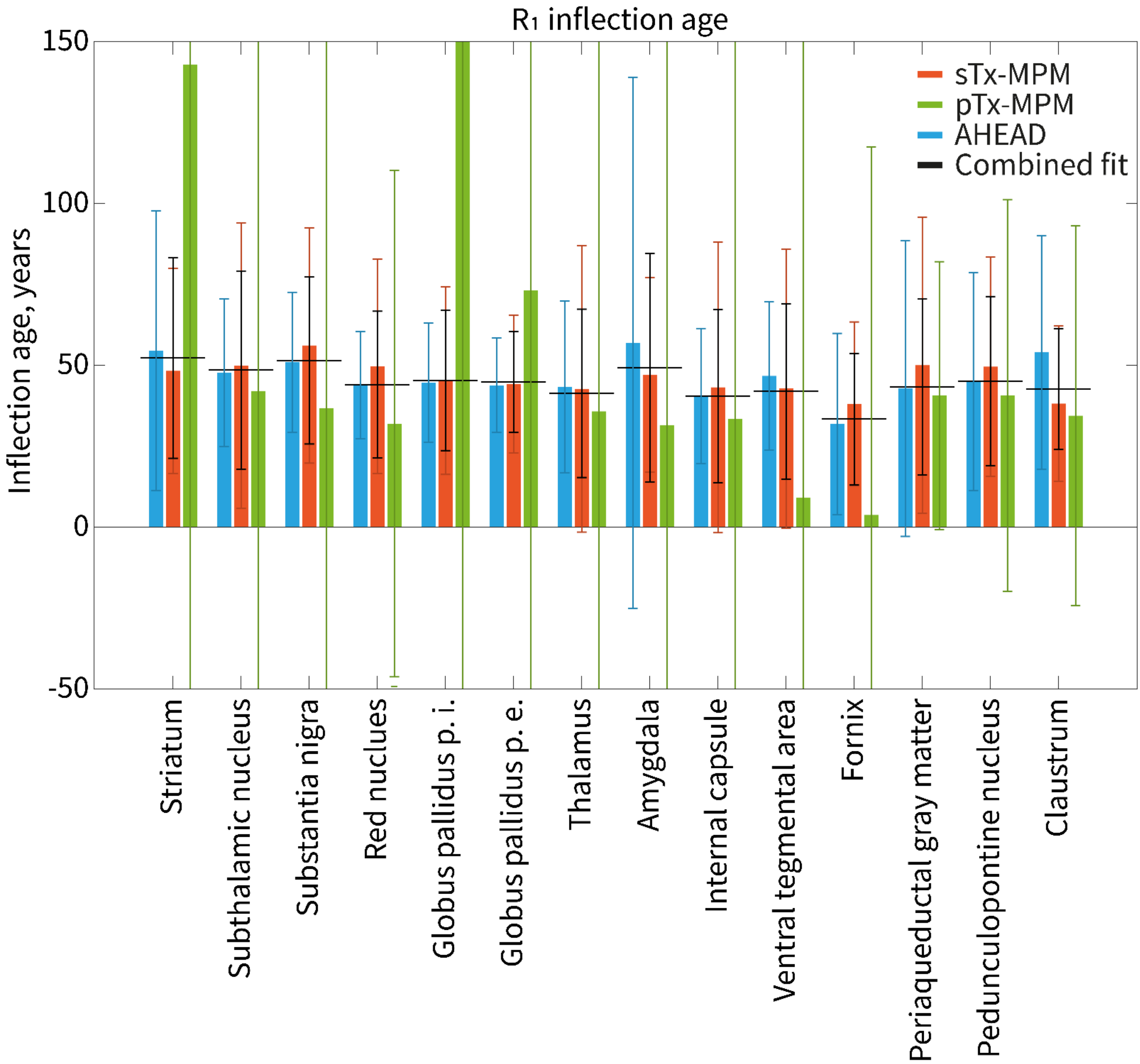

Figure S7 Ages of maximal R1 calculated from the age trajectories of median ROI values. The error in determining the inflection age is extremely high for each individual dataset (particularly for pTx-MPM data, exceeding often any reasonable lifespan). Note, that combining datasets does not always lead to error reduction.

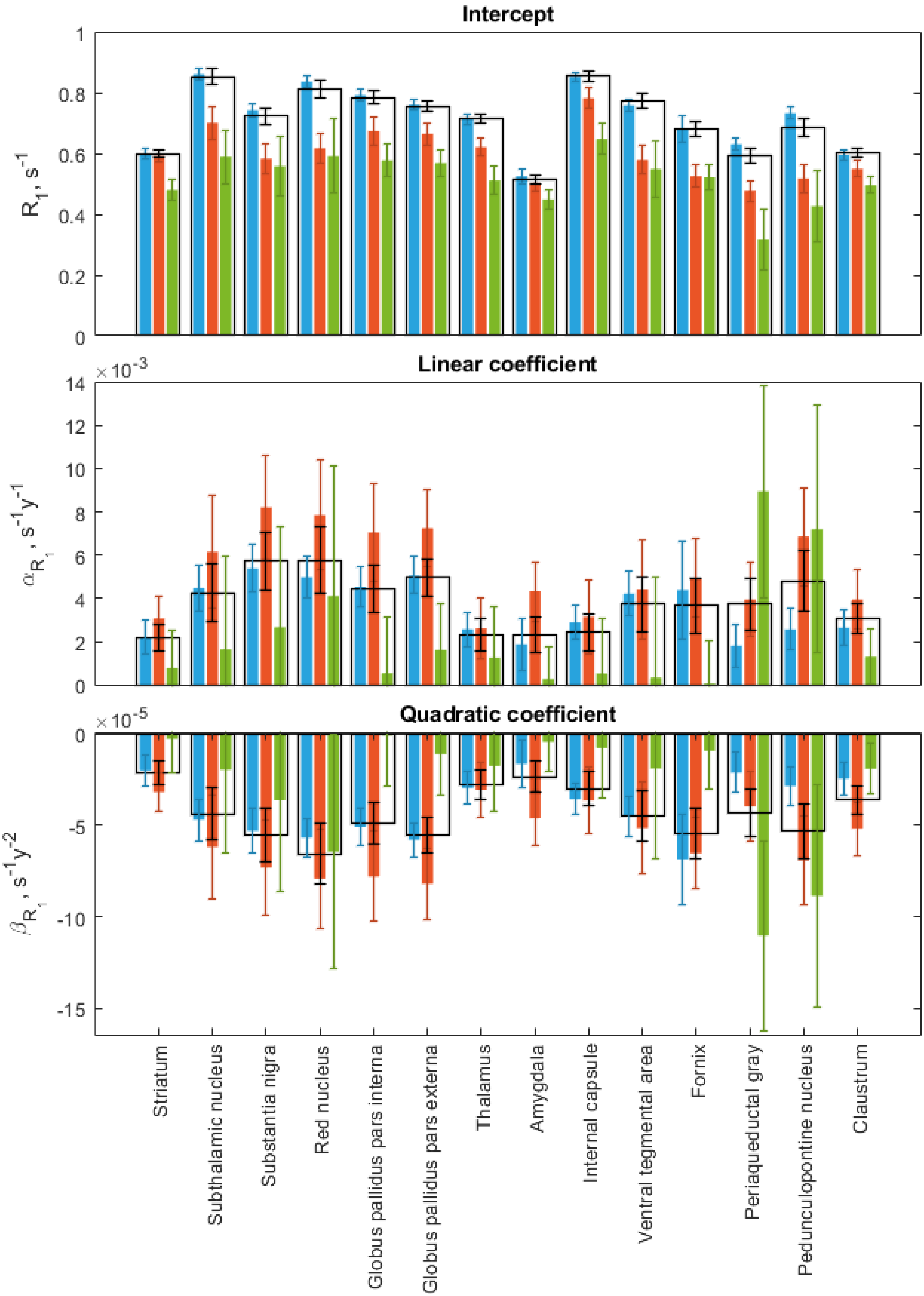

*Figure S8 Age-dependence linear model (metric ~ intercept + α*age + β*age²) coefficients for age dependency of median regional R1 values for different regions. Colored columns show effect size for per-dataset fits (blue for AHEAD data, red for sTx-MPM, green for pTx-MPM), black bars indicate effect sizes for a combined dataset fit.*

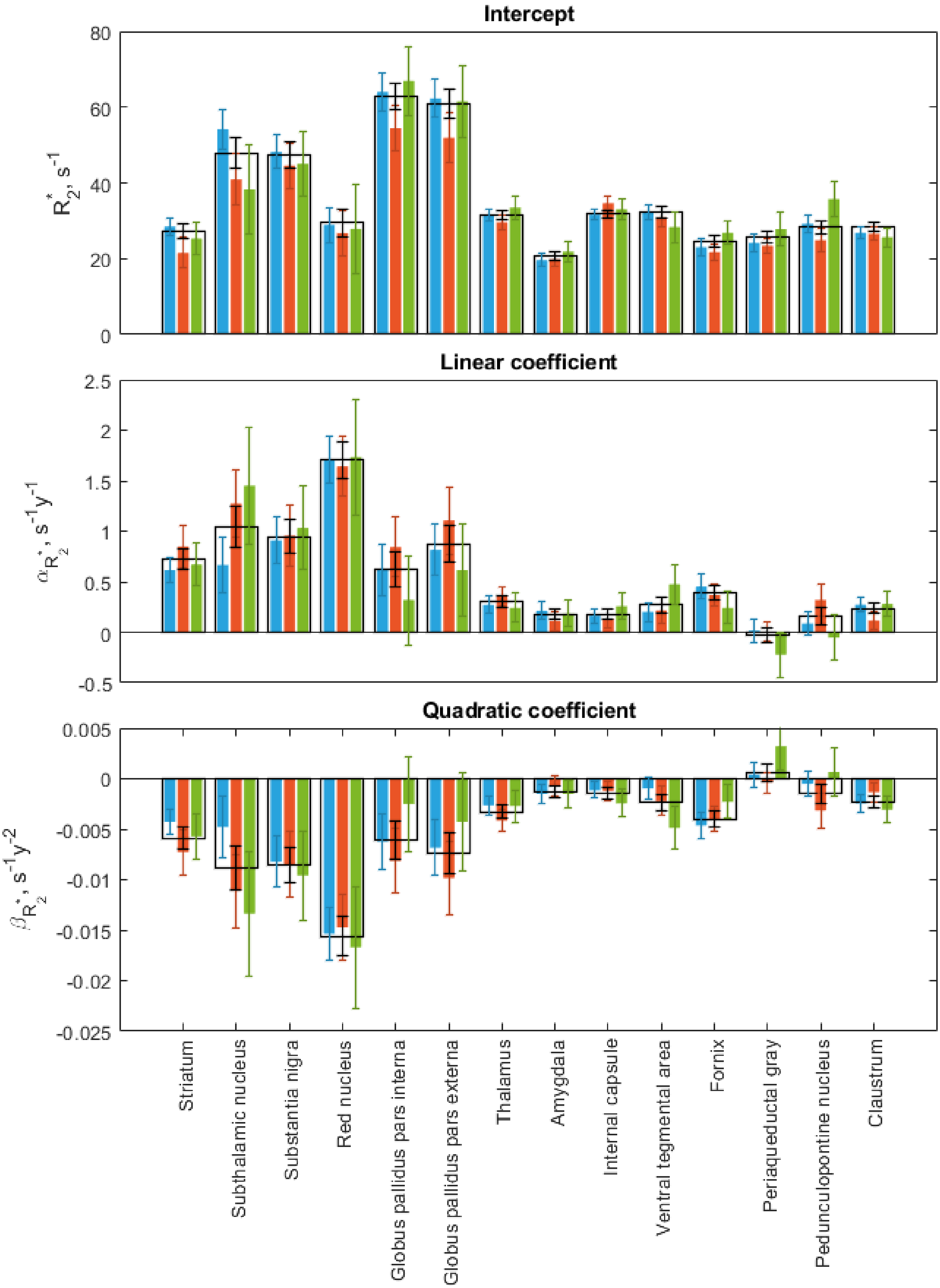

*Figure S9 Age-dependence linear model (metric ~ intercept + α*age + β*age²) coefficients for for age dependency of median regional R2*. Colored columns show effect size for per-dataset fits (blue for AHEAD data, red for sTx-MPM, green for pTx-MPM), black bars indicate effect sizes for a combined dataset fit.*

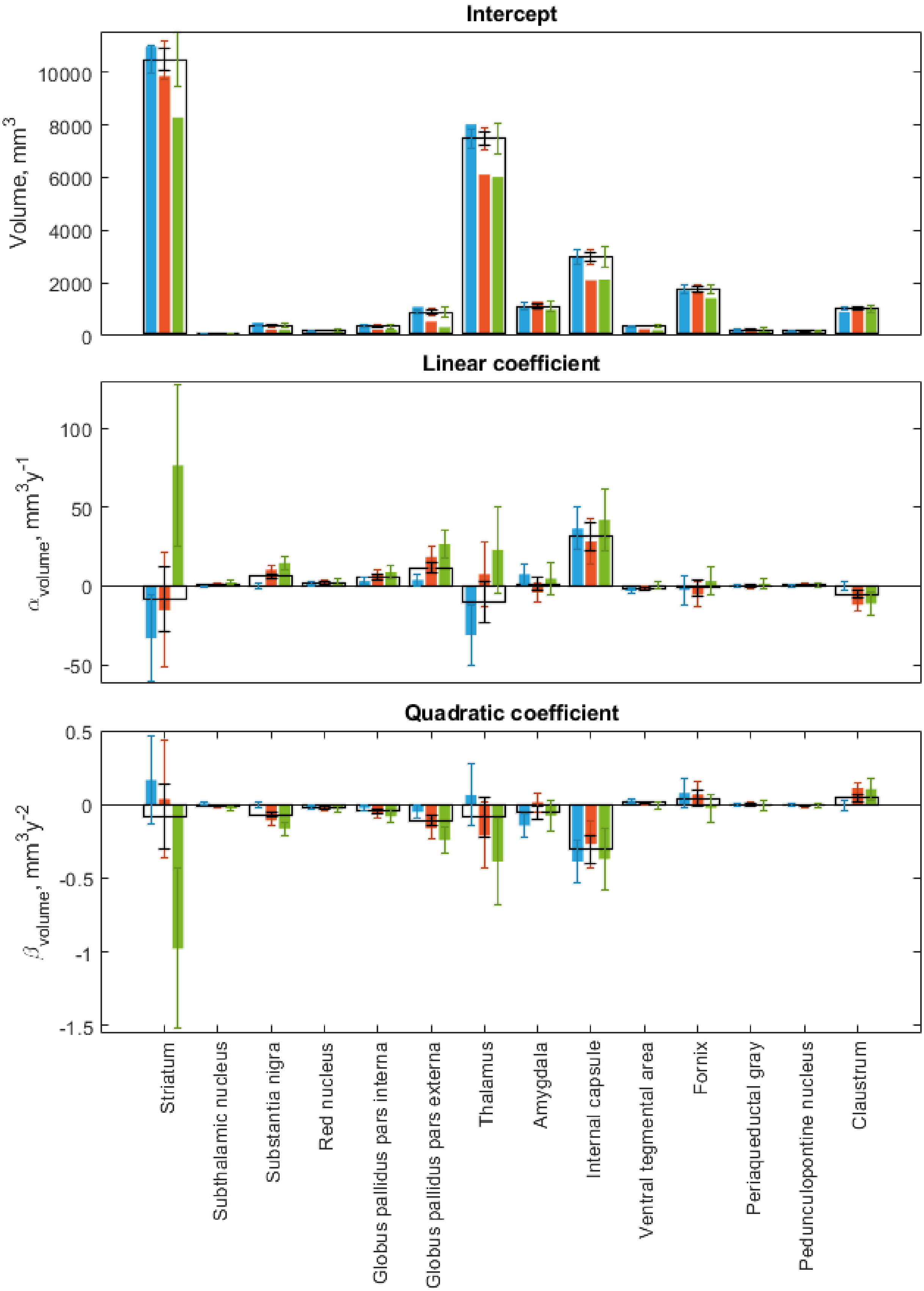

*Figure S10 Age-dependence linear model (metric ~ intercept + α*age + β*age^2) coefficients for ROI volume. Colored columns show effect size for per-dataset fits (blue for AHEAD data, red for sTx-MPM, green for pTx-MPM), black bars indicate effect sizes for a combined dataset fit.*

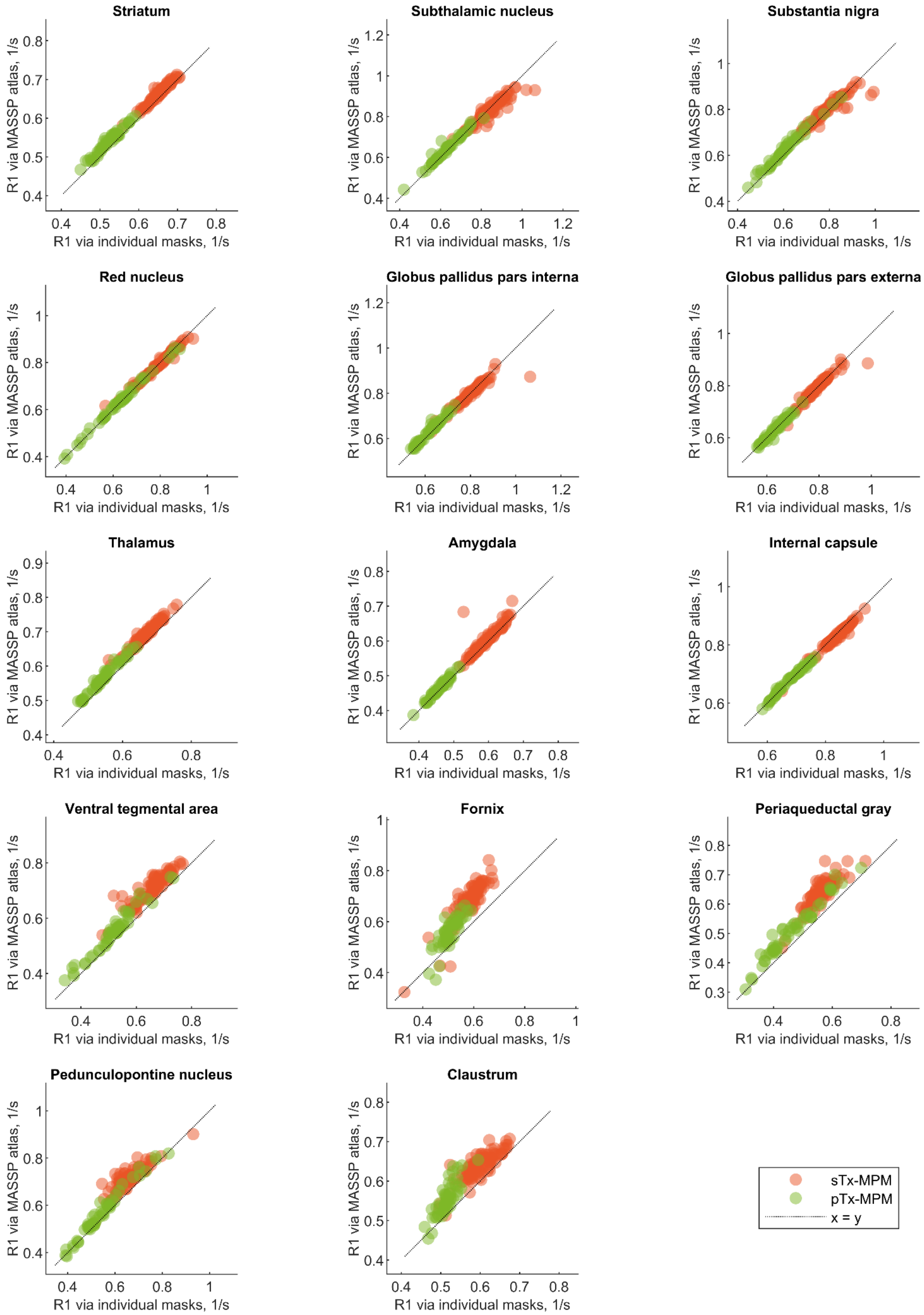

Figure S11Quantifing impact of protocol-specific segmentation on median regional R1 values. Correlation plots between median R1 values in ROIs defined via MAPPS-based segmentation performed on qMRI maps, and ROIs defined via MASSP atlas without the use of qMRI maps for segmentation. The correlation closely follows unity line for most regions, demonstrating relatively small impact of segmentation on regional R1 values, apart from several small regions including subthalamic nuclei, fornix, periaqueductal gray, pedunculopontine nucleus and claustrum.

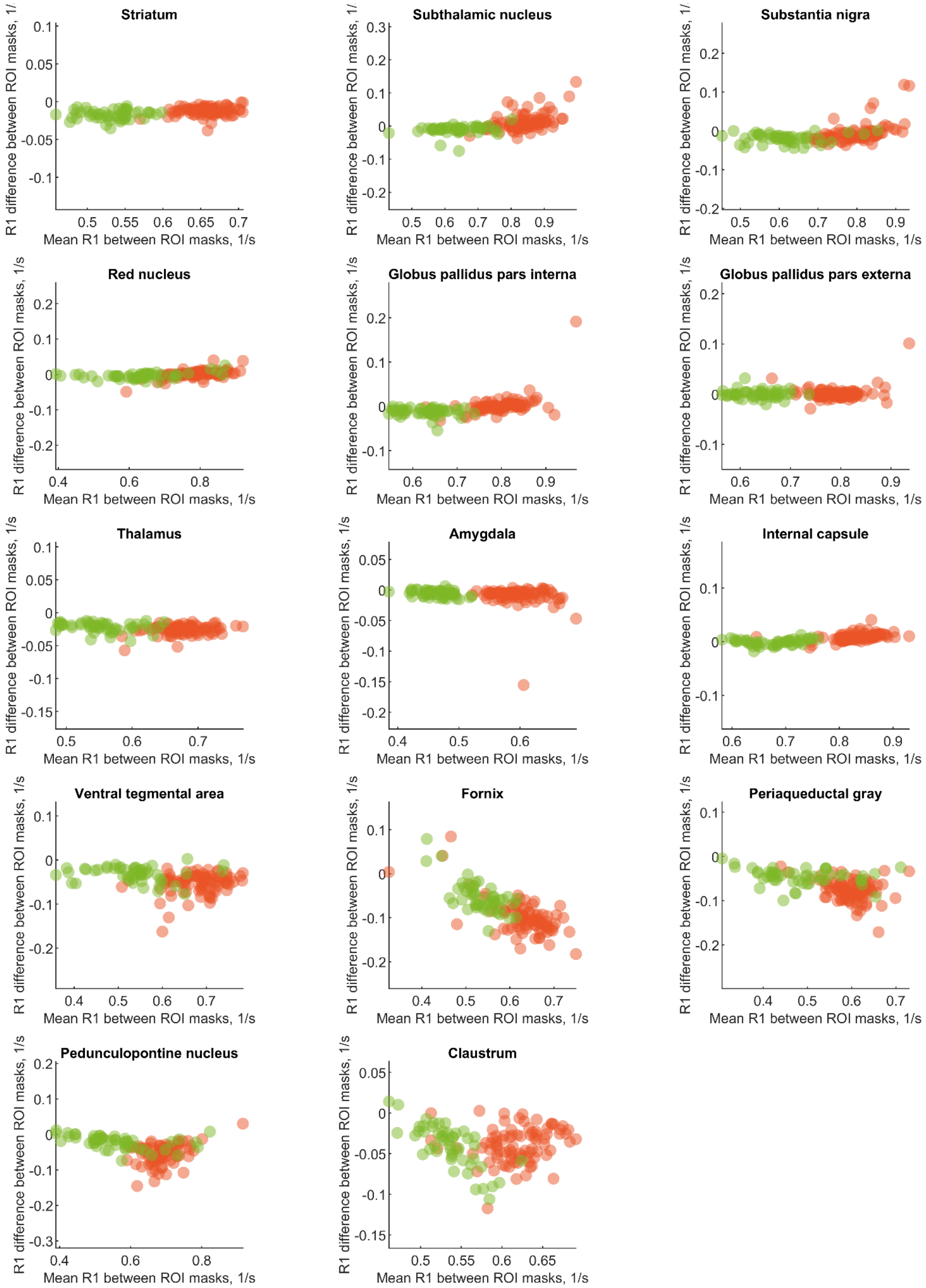

Figure S12 Quantifing impact of protocol-specific segmentation on median regional R1 values. Bland-Altman plots for median R1 values in ROIs defined via MAPPS-based segmentation performed on qMRI maps, and ROIs defined via MASSP atlas without the use of qMRI maps. for segmentation. The bias induced by segmentation is below 5% for most regions, but impacts median values in small regions including subthalamic nuclei, fornix, periaqueductal gray, pedunculopontine nucleus and claustrum. Importantly, the difference in median values induced by segmentation is much smaller compared to difference in R1 values between protocols.

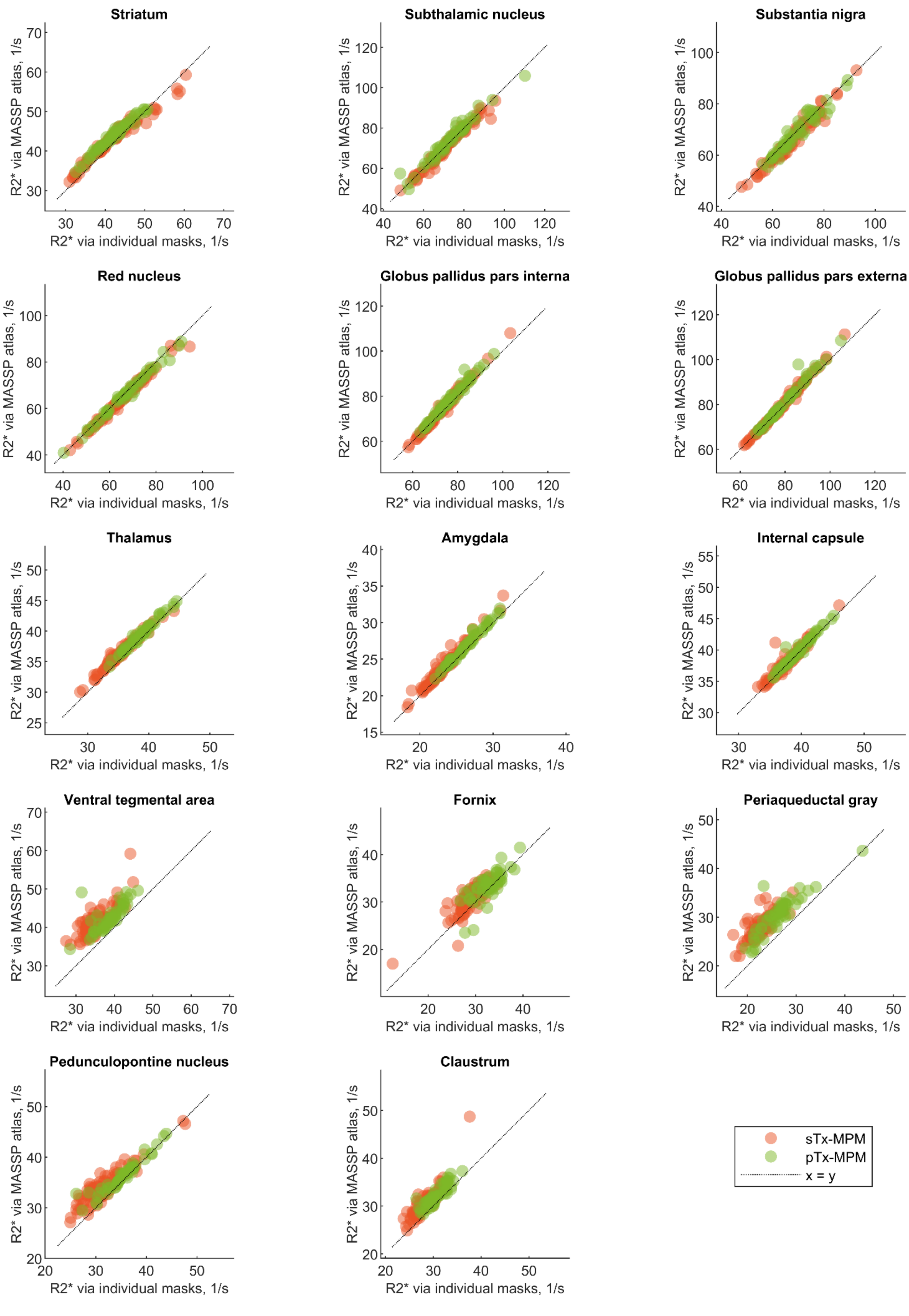

Figure S13 Quantifying impact of protocol-specific segmentation on median regional R2 values. Correlation plots between median R2 values in ROIs defined via MAPPS-based segmentation performed on qMRI maps, and ROIs defined via MASSP atlas without the use of qMRI maps. The correlation closely follows unity line for most regions, demonstrating relatively small impact of segmentation on regional R2 values.*

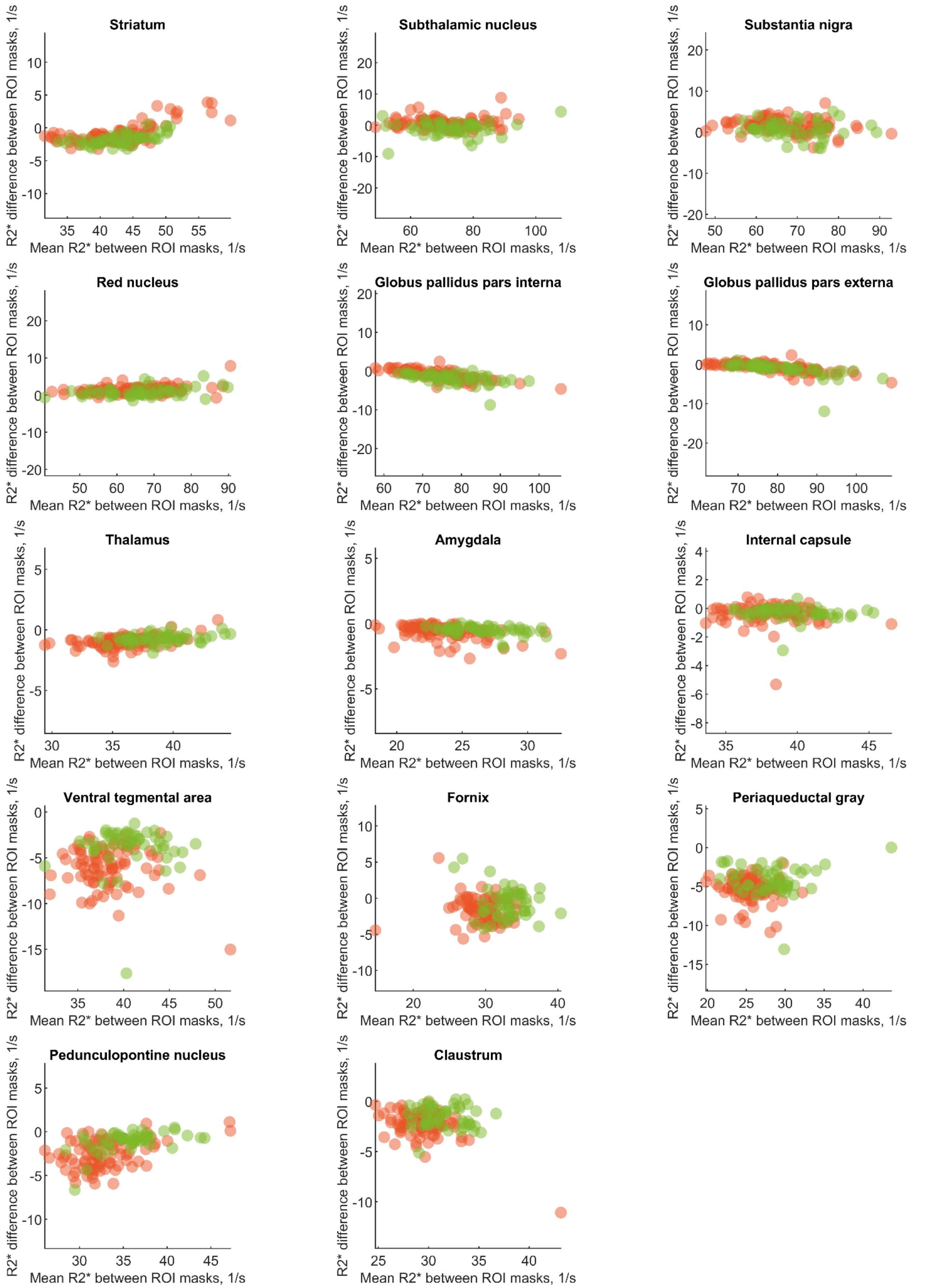

Figure S14 Quantifying impact of protocol-specific segmentation on median regional R2 values. Bland-Altman plots for median R2 values in ROIs defined via MAPPS-based segmentation performed on qMRI maps, and ROIs defined via MASSP atlas without the use of qMRI maps. The bias induced by segmentation is below 10% for most regions, but is comparable to difference in quantitative R2* values between protocols.*

Table S1 *Quantification of the relative impact of protocol-dependent differences in qMRI values and protocol-specific segmentation on median regional R1 values. Segmentation- and quantification-related biases in R1 were compared. For each region, the segmentation-induced bias (columns 2–3) was calculated as the cohort-mean difference between median regional values obtained using subject-specific MASSP segmentation and those obtained using atlas-based ROIs. The bias induced by protocol-dependent qMRI values (column 4) was calculated as the difference between cohort means of ROI medians derived from two protocols using atlas-based ROIs (to ensure a consistent ROI selection approach across protocols). The impact of protocol-dependent qMRI values is much larger for most ROI as compared to the impact of protocol-dependent segmentation.*

R1	Average pTx-MPM segmentation-related bias, p.u.	Average sTx-MPM segmentation-related bias, p.u.	Bias between sTx and pTx averaged medians, p.u.
Striatum	-3.23 %	-1.85 %	-20.46 %
Subthalamic nucleus	-1.81 %	1.31 %	-25.62 %
Substantia nigra	-3.23 %	-1.03 %	-24.16 %
Red nucleus	-0.66 %	0.03 %	-22.23 %
Globus pallidus pars interna	-2.04 %	0.17 %	-22.72 %
Globus pallidus pars externa	0.02 %	0.14 %	-21.77 %
Thalamus	-3.90 %	-3.85 %	-19.92 %
Amygdala	-1.22 %	-1.64 %	-25.57 %
Internal capsule	-0.14 %	1.00 %	-19.98 %
Ventral tegmental area	-6.00 %	-7.91 %	-25.00 %
Fornix	-9.50 %	-15.34 %	-19.52 %
Periaqueductal gray	-9.56 %	-12.83 %	-22.59 %
Pedunculopontine nucleus	-3.63 %	-8.05 %	-22.72 %
Claustrum	-7.32 %	-6.39 %	-13.79 %

Table S2 *Quantification of the relative impact of protocol-dependent differences in qMRI values and protocol-specific segmentation on median regional R2* values. Segmentation- and quantification-related biases in R2 were compared. For each region, the segmentation-induced bias (columns 2–3) was calculated as the cohort-mean difference between median regional values obtained using subject-specific MASSP segmentation and those obtained using atlas-based ROIs. The bias induced by protocol-dependent qMRI values (column 4) was calculated as the difference between cohort means of ROI medians derived from two protocols using atlas-based ROIs (to ensure a consistent ROI selection approach across protocols). The protocol-dependency of R2* values is generally small, but the impact of protocol-dependent qMRI is comparable to the impact of protocol-dependent segmentation. VTA is affected by segmentation bias the most.*

R2*	Average pTx-MPM segmentation-related bias, p.u.	Average sTx-MPM segmentation-related bias, p.u.	Bias between sTx and pTx averaged medians, p.u.
Striatum	-4.18 %	-2.53 %	1.99 %
Subthalamic nucleus	-1.52 %	1.03 %	4.51 %
Substantia nigra	0.97 %	2.58 %	3.90 %
Red nucleus	1.17 %	2.16 %	2.40 %
Globus pallidus pars interna	-2.36 %	-1.10 %	6.27 %
Globus pallidus pars externa	-1.41 %	-0.86 %	3.16 %
Thalamus	-1.88 %	-2.76 %	7.29 %
Amygdala	-1.94 %	-2.73 %	11.94 %
Internal capsule	-0.83 %	-1.02 %	3.81 %
Ventral tegmental area	-9.50 %	-15.76 %	2.88 %
Fornix	-1.95 %	-5.80 %	9.19 %
Periaqueductal gray	-14.89 %	-20.98 %	7.63 %
Pedunculopontine nucleus	-3.14 %	-8.10 %	6.34 %
Claustrum	-4.84 %	-7.67 %	5.27 %
